# Roux-en-Y gastric bypass potentially improved intestinal permeability by regulating gut innate immunity in diet-induced obese mice

**DOI:** 10.1038/s41598-021-94094-8

**Published:** 2021-07-21

**Authors:** Zhangliu Jin, Kai Chen, Zhe Zhou, Weihui Peng, Wei Liu

**Affiliations:** 1 Department of General Surgery, The Second Xiangya Hospital, Central South University, Changsha, Hunan 410011 China; 2 Department of Biliopancreatic and Metabolic Surgery, The Second Xiangya Hospital, Central South University, Changsha, Hunan 410011 China

**Keywords:** Metabolic syndrome, Obesity

## Abstract

Roux-en-Y gastric bypass (RYGB) has been demonstrated to be the most effective treatment for morbid obesity, yet the impact of RYGB on intestinal permeability is not fully known. In this work, we subjected obese mice to RYGB and sham operation procedures. Serum lipopolysaccharide (LPS) level, inflammatory cytokines and intestinal permeability were measured at 8 weeks post surgery. In contrast to sham surgery, RYGB reduced body weight, improved glucose tolerance and insulin resistance, and decreased serum levels of LPS, IL6 and TNFα. Intestinal permeability of the common limb and colon was significantly improved in the RYGB group compared to the sham group. The mRNA levels of IL1β, IL6, and TLR4 in the intestine were significantly decreased in the RYGB group compared with the sham group. The expression levels of intestinal islet-derived 3β (REG3β), islet-derived 3γ (REG3γ) and intestinal alkaline phosphatase (IAP) were higher in the RYGB group than in the sham group. In conclusion, in a diet-induced obesity (DIO) mouse model, both decreased intestinal permeability and attenuated systemic inflammation after RYGB surgery were associated with improved innate immunity, which might result from enhanced production of IAP and antimicrobial peptides.

## Introduction

To date, accumulating evidence has suggested that crosstalk between the microbiota and intestine is essential for body weight homeostasis^1–3^. Intestinal bacteria-derived pathogens play an important role in obesity-related metabolic disorders^4^. The change in microbiota results in an impairment of the gut barrier, which induces the systemic release of lipopolysaccharide (LPS) from the outer membrane of gram-negative bacteria. A wide range of studies have reported that LPS appears to induce chronic low-grade inflammation and regulate fat deposition, which causes insulin resistance and type 2 diabetes mellitus (T2DM)^5,6^.

The gastrointestinal tract confines intestinal bacterial invasion through its physical, chemical and immune barriers while maintaining proper permeability for the absorption of nutrients^7^. A large number of studies have found impairment of the intestinal barrier function in patients with morbid obesity, which mainly presents as increased gut permeability and might lead to systemic inflammation^3,8^. Moreover, intestinal permeability exerted a profound influence on gut-derived inflammation.

Roux-en-Y gastric bypass (RYGB) surgery is the most effective and sustainable treatment for obesity and its comorbidities, such as T2DM and cardiovascular disease^9^. Furthermore, a growing body of literature has revealed that the RYGB procedure improves the state of chronic low-grade inflammation in obesity^10,11^. The alimentary tract is realigned after RYGB, which leads to fundamental changes in nutrient digestion and absorption, the intraluminal environment and intestinal epithelial cells. Moreover, intestinal alkaline phosphatase (IAP) and antimicrobial peptides produced by enterocytes play a pivotal role in neutralizing toxicity from intestinal microbiota as part of the innate immune system and in the subsequent maintenance of intestinal homeostasis^12,13^. Hence, we hypothesized that intestinal permeability was improved by RYGB. In this study, we first report the alteration of intestinal permeability in different intestinal limbs after RYGB in a DIO mouse model, with a mechanistic investigation from the perspective of intestinal inflammation and innate immunity.

## Results

### RYGB surgery reversed HFD-induced obesity and insulin resistance

**Figure 1:**
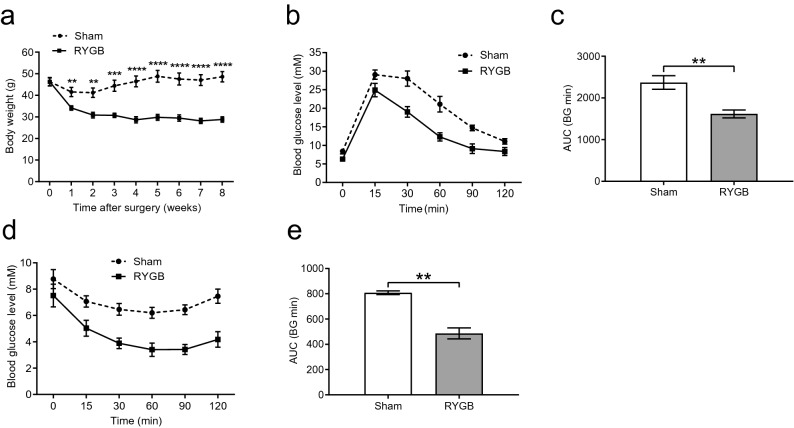
RYGB reduced body weight and improved glucose homeostasis. (**a**) The body weight of mice in the RYGB group (*n* = 5/group) was significantly lower from postoperative week 1 to week 8 than that of mice in the sham group (*n* = 6/group). (**b**,**c**) IPGTT measurement and AUC showed that in contrast to sham surgery, RYGB significantly reduced glucose levels and improved glucose tolerance. (**d**,**e**) The ITT test and AUC revealed that glucose levels were significantly lower in the RYGB group than in the sham group after insulin injection, reflecting enhanced insulin sensitivity. All data are the mean ± SEM. *p < 0.05; **p < 0.01; ***p < 0.005; ****p < 0.001. *AUC* area under curve.

In the RYGB group, one mouse died due to jejunal anastomotic obstruction, and the remaining mice were in good condition; therefore, the operative mortality rate was 16.67%. The body weight of the RYGB group was similar to that of the sham group at the starting point (before the operation). In contrast, the weight loss of mice in the RYGB group was significantly higher than that of mice in the sham group from 1 to 8 weeks postoperatively (Fig. 1a). According to the blood glucose measurement, mice in the RYGB group showed significantly improved glucose tolerance (Fig. 1b,c) and insulin resistance compared to those in the sham group (Fig. 1d,e).

### RYGB surgery restored intestinal barrier integrity

**Figure 2:**
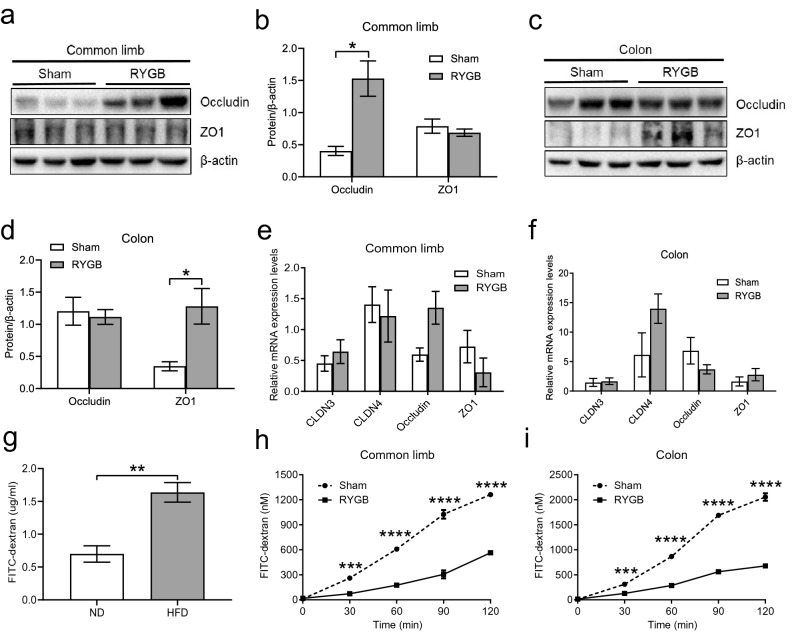
RYGB resulted in the restoration of gut barrier integrity. (**a**,**c**) The levels of TJPs in both the common limb and colon were examined by western blot. (**b**,**d**) Analysis of the gray image of occludin and ZO1 in both the common limb and colon between the sham group (*n* = 6/group) and the RYGB group (*n* = 5/group). (**e**,**f**) Measurement of the mRNA expression of TJPs of both the common limb and colon showed no significant difference between the groups, although the RYGB group (*n* = 5/group) showed a trend of increasing occludin, ZO1 and CLDN4 levels in the common limb and colon, respectively, in comparison to the sham group (*n* = 6/group). (**g**) The comparison of intestinal permeability between the HFD group (HFD, *n* = 5/group) and normal control (ND, *n* = 5/group) was determined by FITC-dextran oral gavage. (**h**,**i**) The intestinal permeability of the sham group and RYGB group was measured by Ussing chamber assay. The levels of FITC-dextran in serosal buffer in both the common limb and colon were significantly lower in the RYGB group (*n* = 4/group) than in the sham group (*n* = 4/group). All data are the mean ± SEM. *CLDN3* claudin-3, *CLDN4* claudin-4, *ZO1* zonula occludens-1. *p < 0.05; **p < 0.01; ***p < 0.005; ****p < 0.001.

The expression levels of tight junction proteins (TJPs), occludin in the common limb (Fig. 2a) and ZO1 in the colon (Fig. 2c), were significantly upregulated in the RYGB group compared to those in the sham group (Fig. 2b,d). Although the mRNA expression levels of certain TJPs in the RYGB group showed a tendency toward elevation in contrast to those in the sham group, the difference was not significant (Fig. 2e,f). By FITC-dextran oral gavage, we demonstrated that high-fat feeding induced impairment of gut permeability (Fig. 2g). However, the intestinal permeability of the common limb (Fig. 2h) and colon (Fig. 2i) was restored in the RYGB group compared to the sham group, according to the Ussing chamber measurement.

### RYGB surgery attenuated systemic and intestinal inflammation

**Figure 3:**
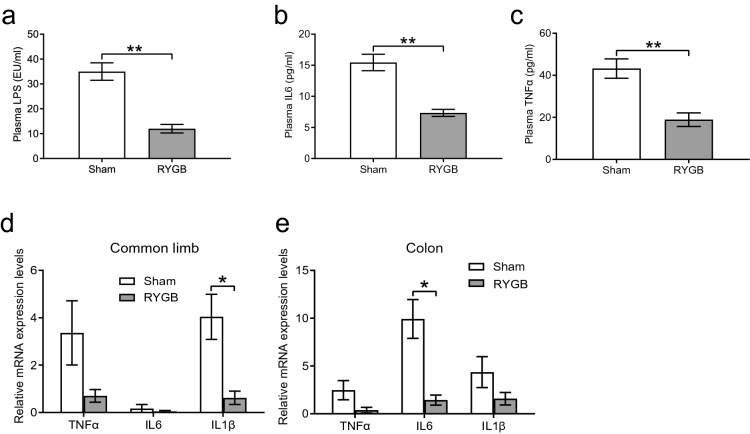
RYGB suppressed systemic and intestinal inflammation. (**a**–**c**) Compared to those in the sham group (*n* = 6/group), levels of plasma inflammatory cytokines, such as LPS, IL6, and TNFα, were notably decreased in the RYGB group (*n* = 5/group). (**d**,**e**) The mRNA levels of inflammatory cytokines in the common limb and colon were determined by quantitative real-time PCR. Compared to the sham group, gut inflammation was reduced after RYGB surgery. *TNFα* tumor necrosis factor-α, *IL6* interleukin-6, *IL1β* interleukin-1β,* LPS* lipopolysaccharide. All data are the mean ± SEM. *p < 0.05; **p < 0.01.

Compared to those in the sham group, the plasma levels of LPS (Fig. 3a), IL6 (Fig. 3b) and TNFα (Fig. 3c) were found to be significantly reduced in the RYGB group, indicating that endotoxemia and systemic inflammation were prominently alleviated by RYGB. Regarding intestinal inflammation, the mRNA expression levels of IL1β in the common limb (Fig. 3d) and IL6 in the colon (Fig. 3e) were significantly lower in the RYGB group than in the sham group, suggesting that RYGB attenuated the inflammatory response of the intestine.

### Restoration of intestinal integrity after RYGB is associated with a decreased TLR4 level in the intestine

**Figure 4:**
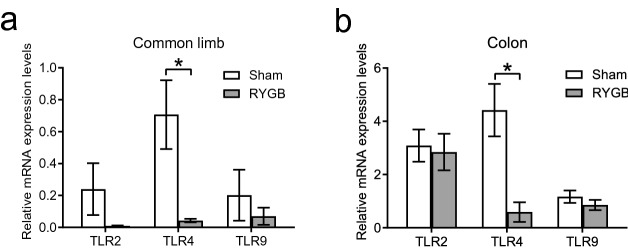
RYGB altered the expression levels of TLRs in the intestine. (**a**,**b**) Quantitative real-time PCR analysed the mRNA levels of TLRs. The mRNA expression levels of TLR4 were markedly downregulated in the common limb and colon of mice in the RYGB group (*n* = 5/group) compared to the sham group (*n* = 6/group). *TLR2* toll-like receptor-2, *TLR4* toll-like receptor-4, *TLR9* toll-like receptor-9. All data are the mean ± SEM. *p < 0.05.

The mRNA expression levels of toll-like receptors (TLRs) showed that the expression of TLR4 in the common limb and colon (Fig. 4a,b) was significantly reduced in the RYGB group compared to the sham group. Although the mRNA levels of TLR2 and TLR9 in the common limb and colon presented a decreasing trend in the RYGB group, the difference did not reach significance.

### RYGB surgery enhanced the production of antimicrobial peptides and IAP from the intestine

**Figure 5:**
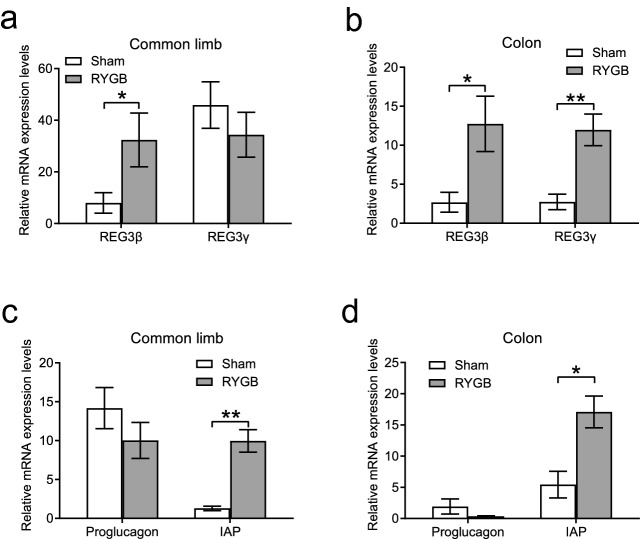
The production of intestinal antimicrobial peptides and IAP was enhanced after RYGB. (**a**,**b**) The mRNA levels of REG3β were significantly upregulated in both the common limb and colon of mice in the RYGB group (*n* = 5/group) compared to the sham group (*n* = 6/group), and the mRNA expression level of REG3γ was  increased in the colon of the RYGB group. (**c**,**d**) The mRNA expression level of IAP, but not proglucagon, was significantly increased in both the common limb and colon. *REG3β* regenerating islet-derived-3 beta, *REG3γ* regenerating islet-derived-3 gamma, *IAP* intestinal alkaline phosphatase. All data are the mean ± SEM. *p < 0.05; **p < 0.01.

We found that the mRNA expression levels of antimicrobial peptides and IAP were augmented after RYGB surgery. In contrast to the sham group, REG3β mRNA levels in the common limb and the colon were markedly increased in the RYGB group (Fig. 5a,b), and the REG3γ mRNA expression level in the colon was significantly higher in the RYGB group than in the sham group (Fig. 5b). Moreover, the mRNA expression of IAP in both the common limb and colon was markedly enhanced by RYGB (Fig. 5c,d).

## Discussion

To our knowledge, this study is the first report on the change in intestinal permeability after RYGB in a DIO mouse model. Our data showed that gut permeability was restored by RYGB in comparison to sham surgery, with marked amelioration of intestinal and systemic inflammation. In mice subjected to RYGB surgery, the small bowel was surgically partitioned into three segments, of which the alimentary and biliopancreatic limbs functioned in the transport of undigested food and digestive juice, respectively^14^. As a result, macronutrient digestion and absorption mainly occur within the common limb after RYGB, which might lead to profound changes in the intraluminal milieu within the common limb and colon. Therefore, our investigation mainly focused on the common limb and colon after RYGB.

In the present study, we showed that RYGB led to significant weight loss and remission of insulin resistance, demonstrating a successful mouse model of RYGB (Fig. 1a). After 4 h of fasting, the fasting blood glucose levels of mice in the RYGB group were not different from those in the sham group, which may be due to stress on the mice (Fig. 1d). As chronic inflammation has been proven to play an essential role in insulin resistance and metabolic syndrome^15,16^, we measured inflammatory cytokines in peripheral blood and concluded that RYGB substantially reduced systemic inflammation, as supported by the finding that IL6 and TNFα were decreased in the RYGB group. Moreover, our study found that serum LPS levels were significantly lower in the RYGB group than in the sham group, suggesting that the intestine became less permeable to gut-derived toxins, which is consistent with the findings of relieved endotoxemia after RYGB in human studies^17,18^.

Intriguingly, several studies^19,20^ revealed that there is an association between increased gut permeability and obesity-related metabolic disorders. TJPs play a critical role in maintaining gut permeability and mainly consist of the transmembrane proteins claudens and occludins and the scaffold protein ZO1^21^. Increasing evidence suggests that the disruption of TJPs results in increased gut permeability^21^. Our findings showed that the levels of occludin in the common limb and ZO1 in the colon were upregulated after RYGB surgery compared to sham surgery (Fig. 2b,d). We found that the mRNA levels of TJPs in the common limb and colon were increased after RYGB, although the difference was not significant. The difference between the mRNA and protein levels may be due to the change in translation and/or the small size of the sample. By FITC-dextran oral gavage, we showed that a HFD was pathogenic for the impairment of gut permeability (Fig. 2g). Due to surgical reconfiguration of the gastrointestinal tract after RYGB, the physiological functions of the common limb and colon might be different from their presurgical functions; hence, we precisely determined the gut permeability of each segment with the Ussing chamber. Based on the findings from the Ussing chamber assays, we revealed that intestinal permeability was restored in the common limb and colon after RYGB. Hence, we found that the upregulation of tight junction proteins could contribute to the restoration of gut permeability (Fig. 2h,i). Aggravated gut permeability persistently leads to the development and progression of chronic inflammation, which causes insulin resistance in obesity^22^. Collectively, our findings indicate that the improved insulin resistance may be related to the restoration of gut permeability after RYGB surgery.

Studies have found excessive inflammation in the gastrointestinal tract of HFD-induced obese mice and patients with morbid obesity^3,23^. Moreover, intestinal permeability may have an impact on their inflammatory status. IL6, IL1β and TNFα have been widely used to assess the severity of gut inflammation^3^. Our data showed a significant reduction in inflammatory factors, such as IL1β and IL6, in the common limb and colon, respectively. Furthermore, because the gastrointestinal tract is colonized by various microorganisms, including numerous species of bacteria, the intestinal barrier can efficiently protect against the disruption of the gut derived from gut microbiota^24^. Altogether, we speculate that the improvement of gut-derived and systemic inflammation may be attributable to the restoration of intestinal permeability in the RYGB group compared to that in the sham group.

A growing body of studies in both human and rodent models have unveiled that obesity and consumption of the western diet are related to the impairment of gut permeability^25–27^. As expected, compared to that of mice fed normal chow, the gut permeability of mice fed a HFD was significantly increased (Fig. 2g). Furthermore, our previous study^28^ indicated that the daily food intake of mice in the RYGB group was not different from that of mice in the sham group. Hence, the amount of food intake is not a confounding factor for the improved gut permeability in the RYGB model. The crosstalk between the microbiota and gut plays a pivotal role in regulating intestinal function and permeability^29^. A large body of evidence has revealed that a close physiological and pathological connection exists between intestinal innate immunity and gut inflammatory stress^30,31^. As a consequence, to define the mechanism by which RYGB mitigates enteric and systemic inflammation, we assessed the innate immune system of the intestine. Our data demonstrated that the mRNA expression of TLR4 was significantly decreased in the common limb and colon following RYGB, suggesting that the intestinal inflammation caused by the LPS-TLR4 pathway was diminished by RYGB.

IAP, a gut brush border enzyme that can dephosphorylate LPS lipid A to detoxify LPS, is secreted into the intestinal lumen by enterocytes, modulates mucosal inflammation via the TLR4 pathway^32^ and maintains gut homeostasis^33^. We found that intestinal IAP production was significantly strengthened by RYGB in the common limb and colon, suggesting that the LPS-TLR4 inflammatory pathway might be downregulated through enhanced IAP secretion. Moreover, intestinal epithelial cells can also generate antimicrobial peptides such as REG3β and REG3γ, which play a critical role in suppressing gut gram-positive bacteria^34,35^. This study revealed that the expression of REG3β and REG3γ was markedly increased in the common limb and colon after RYGB surgery, which may assist in inhibiting gut-derived inflammation and improve gut permeability^36,37^. Taken together, these findings indicate that augmented production of IAP and antimicrobial peptides may exert protective effects against intestinal inflammatory stress after RYGB, which results in the improvement in gut permeability.

In terms of the limitations of the study, we found an association between intestinal permeability and systemic inflammation, but the causal relationship needs to be further investigated. In addition, we found an improvement in intestinal permeability and alterations in related inflammatory cytokines after RYGB in rodent experiments. Whether this result applies to humans is unclear; therefore, we will further verify these data in clinical patients in the future.

## Conclusion

In this study, we first demonstrated that RYGB improved gut permeability in a DIO mouse model and unveiled the association between improved intestinal permeability and enhanced production of IAP and antimicrobial peptides. This study will help to elucidate the mechanism of RYGB and facilitate our understanding of the crosstalk between the gut and obesity.

## Methods

### Experimental animals

Twelve seven-week-old male C57BL/6 mice were purchased from SLAC Laboratory Animal Inc. (Shanghai, China). All animals received water and normal chow (SLAC, Laboratory Animal Inc., Shanghai, China) ad libitum after birth. At the age of 8 weeks, all animals were fed a high-fat diet (HFD) (60 kcal% fat, #D12492; Research Diets Inc., New Brunswick, NJ, USA) for 12 weeks and subsequently allocated to two groups (sham operation and RYGB surgery). Note that animals in these two groups had similar baseline body weights before surgery. All mice were housed under a 12-h light/12-h dark cycle at room temperature (21 ± 2 °C). The procedures for all animal investigations were performed according to Central South University guidelines for the use of animals, with the approval of the Institutional Animal Care and Use Committee of Central South University. This study was carried out in compliance with the ARRIVE guidelines.

### Preoperative preparation

All animals were randomly allocated into two groups: the RYGB group (RYGB surgery, *n* = 6) and the sham group (sham operation, *n* = 6). All animals were fasted overnight presurgery. The surgical procedures were performed under anesthesia with isoflurane (3–4% for induction and 1–2% for maintenance).

### Surgery

All animal surgeries were performed under an approved surgical model protocol^38^. In brief, a 1.0-cm midline incision was made on the upper abdomen. For mice in the RYGB group, the jejunum was transected 4.0 cm distal to the Treitz ligament, and the proximal part was the biliopancreatic limb. Four centimeters of the distal part was measured and designated as the alimentary limb and then anastomosed to the biliopancreatic limb in an end-to-side fashion by interrupted suture. The gastroesophageal junction was ligated to exclude the stomach, and then a longitudinal incision of 0.2 cm was made on the distal esophagus. The alimentary limb was anastomosed to the distal esophagus in a side-to-side pattern by running sutures. The abdominal incision was closed, and postoperative care was administered. Mice in the sham group underwent jejunal transection and reanastomosis in situ, and the operating time was extended to be the same as that of the RYGB group.

### Postoperative care

After surgery, all animals were placed on a heating pad after injection of 1.0 ml 0.9% saline and 1.0 mg/kg buprenorphine. All mice received a liquid diet (ENSURE, Abbott, Zwolle, Netherlands) for 48 h after surgery and were then switched to a HFD.

### Body weight, IPGTT, and ITT

Mouse body weight was measured weekly at 11:00 a.m.–12:00 a.m. to the nearest 0.1 g. After the mice were fasted for 16 h in postoperative week 7, an intraperitoneal glucose tolerance test (IPGTT) was performed following administration of a glucose solution (1.5 g/kg body weight) by intraperitoneal injection. An insulin tolerance test (ITT) was conducted with intraperitoneal injection of insulin at 0.75 U/kg body weight after 4 h of fasting. At 0, 15, 30, 60, 90 and 120 min after injection, blood was retrieved from the tail vein for blood glucose measurement using an ACCU-CHEK Performa glucometer (Roche, Mannheim, Germany).

### Plasma IL-6, TNFα and LPS

Mice were euthanized at 8 weeks post surgery, followed by the collection of blood and tissue samples. Plasma IL6 and TNFα levels were measured with the relevant enzyme-linked immunosorbent assay kits (BiomeTech, Greifswald, Germany), and plasma LPS concentrations were assessed using the limulus amebocyte lysate (LAL) test (Associates of Cape Cod, East Falmouth, MA, USA).

### Western blot

Gut tissues were homogenized in lysis buffer and sonicated for 1 min. Proteins were quantified by the microplate BCA method. Proteins were separated by 8% or 10% sodium dodecyl sulfate–polyacrylamide gel electrophoresis and transferred to polyvinylidene fluoride (PVDF) membranes (Millipore, USA). The membranes were blocked for 1 h in 2% BSA and subsequently incubated in primary antibody overnight at 4 °C with shaking. Then, the membranes were washed with TBS/Tween and placed in the appropriate secondary antibody in blocking buffer for 1 h at room temperature with shaking. After washing, bound antibodies were visualized using a chemiluminescence detection system. Protein levels were analyzed by densiometric measurements from ImageJ Software. Antibodies against occludin and zonula occludens-1 (ZO1) were purchased from Proteintech (Chicago, IL, USA). All protein levels were normalized to the level of β-actin (Sigma, St. Louis, MO, USA).

### RNA extraction and quantitative real-time PCR

The intestine of RYGB mice was divided into four segments after RYGB, including the alimentary limb, biliopancreatic limb, common limb and colon, which were rapidly frozen in liquid nitrogen after sampling and then stored at − 80 °C for further analysis. The common limb of RYGB mice was equivalent to the lower small bowel of sham mice. RNA was extracted from snap-frozen specimens with TRIzol reagent (Invitrogen, CA, USA). Reverse transcription of RNA was performed using cDNA synthesis kits (Thermo Fisher Scientific, NY, USA). SYBR green dye was used as a fluorochrome for quantitative real-time PCR (qPCR) in the ViiA7 System (Life Technology). The mRNA levels in the intestine were measured by qPCR and normalized to β-actin. The specific primers used are listed in Table S1.

### Intestinal permeability assay in vivo

This experiment was based on intestinal permeability to 4 kDa FITC-dextran from Sigma. Mice were fasted for 4 h and then administered FITC-dextran by oral gavage (600 mg/kg body weight). After 4 h, blood samples were prepared. The blood was centrifuged at 4 °C and 13,000 rpm for 10 min, and the serum was separated. The serum fluorescence intensity of each sample was assessed (excitation: 485 nm, emission: 535 nm) with a fluorescence spectrophotometer (Spark 10 M, Tecan, Switzerland). The FITC-dextran concentrations in the samples were calculated from a standard curve (Fig. S1).

### Ussing chamber experiments and intestinal permeability assay of the common limb and colon

Intestinal permeability was examined 8 weeks after surgery. Briefly, for the Ussing chamber experiment, it is essential to keep the excised intestinal tissue fresh. The intestinal muscle layer was stripped off, and the mucosa was mounted in an Ussing chamber (WPI Instruments, Sarasota, FL, USA) containing 5 ml of circulating oxygenated Krebs buffer (136 mM NaCl, 4.3 mM KCl, 1.5 mM CaCl_2_, 1.4 mM MgSO_4_, 27 mM NaHCO_3_, 1.6 mM KH_2_PO_4_, pH 7.30)^39^. The mucosal-to-serosal flux level of dextran conjugated to fluorescein isothiocyanate (FITC-dextran, molecular weight: 4 kDa, Sigma, MO, USA) was expressed as the intestinal permeability. After the dextran probe was added, specimens of serosal buffer were retrieved at 0, 30, 60, 90 and 120 min and then Krebs buffer/glucose was added. With respect to data analysis, the concentration was estimated in accordance with a standard curve.

### Statistical analysis

*SPSS* 21.0 software (*SPSS* Inc., Chicago, IL, USA) and GraphPad Prism 8.4.2 (GraphPad Software Inc., San Diego, CA, USA) were used to analyze all results. Statistical analysis of the data was performed by Student’s t-test or ANOVA. All data are shown as the mean ± SEM. *p* ≤ 0.05 was considered to be statistically significant.

## Supplementary Information

Supplementary Information.
