# Supplementary material for: Roux-en-Y gastric bypass potentially improved intestinal permeability by regulating gut innate immunity in diet-induced obese mice
Source: Sci Rep. 2021 Jul 21;11:14894. doi: 10.1038/s41598-021-94094-8 (PMC8295358; doi:10.1038/s41598-021-94094-8)

Supplementary Information

Roux-en-Y Gastric Bypass Potentially Improved Intestinal Permeability by Regulating Gut Innate Immunity in Diet-Induced Obese Mice

Zhangliu Jin, Kai Chen, Zhe Zhou, Weihui Peng, Wei Liu*

Department of General Surgery, The Second Xiangya Hospital, Central South University, Changsha, Hunan 410011, China

Department of Biliopancreatic and Metabolic Surgery, The Second Xiangya Hospital, Central South University, Changsha, Hunan 410011, China

***Corresponding Author**

Wei Liu, MD, Professor, Department of General Surgery, Department of Biliopancreatic and Metabolic Surgery, The Second Xiangya Hospital, Central South University, No. 139 Renmin Road, Changsha City, Hunan Province, 410011, China.

Email Address: liuweixy@csu.edu.cn, Phone number: 86 731 85295821 (O) 86 731 8529 5535 (Fax)

Figure S1 The FITC-dextran standard curve was established between HFD group and normal control group.

Figure S1

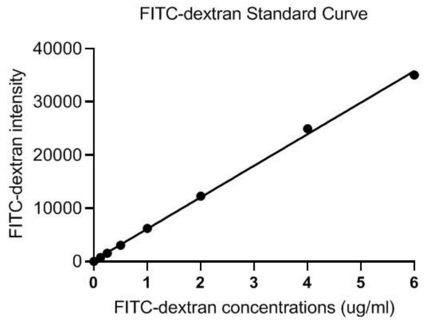

Table S1: Primer pairs sequences

Gene	Primer	Sequence
TNFα	Forward primer	5'- GGTGCCTATGTCTCAGCCTCTT -3'
	Reverse primer	5'- GCCATAGAACTGATGAGAGGGAG -3'
IL6	Forward primer	5'- TACCACTTCACAAGTCGGAGGC -3'
	Reverse primer	5'- CTGCAAGTGCATCATCGTTGTTC-3'
IL1β	Forward primer	5'- TGGACCTTCCAGGATGAGGACA -3'
	Reverse primer	5'- GTTCATCTCGGAGCCTGTAGTG -3'
CLDN3	Forward primer	5'- TCATCGTGGTGTCCATCCTGCT-3'
	Reverse primer	5'- AGAGCCGCCAACAGGAAAAGCA-3'
CLDN4	Forward primer	5'- CGAGCCCTTATGGTCATCAGCA-3'
	Reverse primer	5'- ATGCTTGCCACGATGAACACGG-3'
Occludin	Forward primer	5'- TGGCAAGCGATCATACCCAGAG -3'
	Reverse primer	5'- CTGCCTGAAGTCATCCACACTC -3'
ZO1	Forward primer	5'- GTTGGTACGGTGCCCTGAAAGA-3'
	Reverse primer	5'- GCTGACAGGTAGGACAGACGAT-3'
TLR2	Forward primer	5'- ACAGCAAGGTCTTCCTGGTTCC-3'
	Reverse primer	5'- GCTCCCTTACAGGCTGAGTTCT-3'
TLR4	Forward primer	5'- AGCTTCTCCAATTTTTCAGAACTTC-3'
	Reverse primer	5'- TGAGAGGTGGTGTAAGCCATGC-3'
TLR9	Forward primer	5'- GCTGTCAATGGCTCTCAGTTCC-3'
	Reverse primer	5'- CCTGCAACTGTGGTAGCTCACT-3'
REG3β	Forward primer	5'- TGG GAA TGG AGT AACAAT G -3'
	Reverse primer	5'- GGCAACTTCACC TCACAT -3'
REG3γ	Forward primer	5'- CCA TCTTCACGTAGCAGC -3'
	Reverse primer	5'- CAA GAT GTCCTGAGGGC -3'
Proglucagon	Forward primer	5'- GACATGCTGAAGGGACCTTTAC -3'
	Reverse primer	5'- GGCTTTCACCAGCCAC -3'
IAP	Forward primer	5'- CCAGCAGTAACTCACCTCATGG -3'
	Reverse primer	5'- GAAGCCTTGTGGATTCCTGCTG -3'
β-actin	Forward primer	5'- CATTGCTGACAGGATGCAGAAGG -3'
	Reverse primer	5'- TGCTGGAAGGTGGACAGTGAGG -3'

Original western blot

The catalog of molecular weight marker performed in the experiment was No. 26616. The image acquisition of the strip was done using the ChemiDoc XRS+ Imaging System. Because the ileum segment of the sham group was equivalent to the common limb of the RYGB group, the strip of the common limb was labeled the ileum (Figure 2a).

Figure 2a

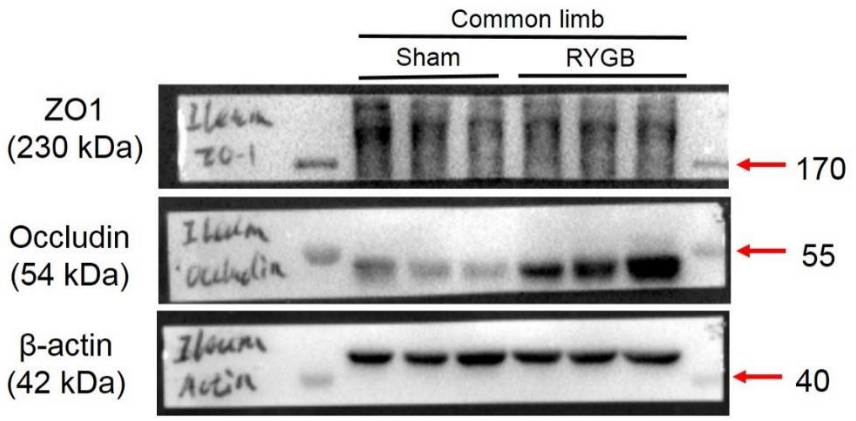

Figure 2c

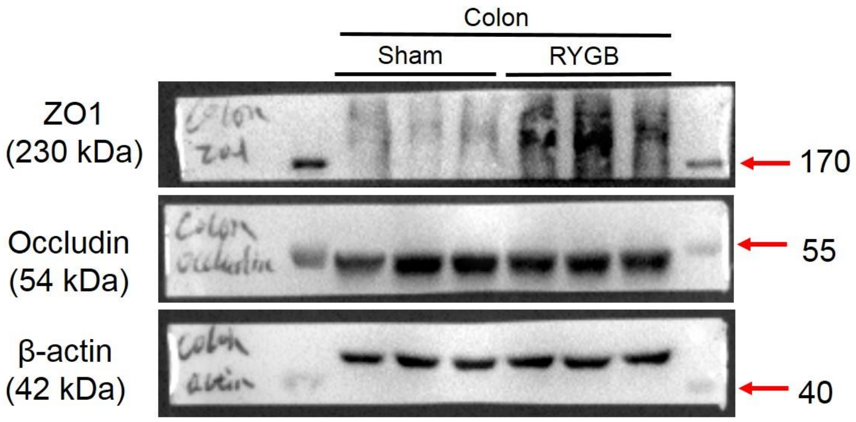

Supplement: Supplementary file 1 — Supplementary Information. [file 41598_2021_94094_MOESM1_ESM.pdf]
